# Partner Weight Loss and Diet Outcomes at 24 Months in a Randomized Controlled Trial of a Dyadic Versus Individual Weight Management Intervention

**DOI:** 10.1002/osp4.70178

**Published:** 2026-08-12

**Authors:** Kristen E. Gray, Laura S. Porter, Ryan J. Shaw, Kara L. Gavin, Scott J. Hetzel, Megan A. Lewis, Samantha Pabich, Heather M. Johnson, Felix Elwert, Lu Mao, Katya Garza, William S. Yancy, Corrine I. Voils

**Affiliations:** ^1^ Health Systems Research VA Puget Sound Health Care System Seattle Washington USA; ^2^ Department of Health Systems and Population Health University of Washington Seattle Washington USA; ^3^ Department of Psychiatry & Behavioral Sciences Duke School of Medicine Durham North Carolina USA; ^4^ Duke University School of Nursing Durham North Carolina USA; ^5^ Department of Medicine School of Medicine and Public Health University of Wisconsin Madison Wisconsin USA; ^6^ Department of Biostatistics and Medical Informatics School of Medicine and Public Health University of Wisconsin Madison Wisconsin USA; ^7^ RTI International Research Triangle Park North Carolina USA; ^8^ William S Middleton Memorial Veterans Hospital Madison Wisconsin USA; ^9^ Baptist Health South Florida/Charles E. Schmidt College of Medicine Florida Atlantic University Boca Raton Florida USA; ^10^ Department of Sociology, Biostatistics and Medical Informatics, Population Health Sciences University of Wisconsin Madison Wisconsin USA; ^11^ Department of Kinesiology University of Wisconsin‐Madison School of Education Madison Wisconsin USA; ^12^ Department of Medicine Duke University School of Medicine Durham North Carolina USA; ^13^ Department of Internal Medicine Spencer Fox Eccles School of Medicine University of Utah Salt Lake City Utah USA

**Keywords:** behavioral therapies, obesity, randomized controlled trial, social support

## Abstract

**Background:**

Including romantic partners as supports in behavioral weight management programs targeting index participants could yield spillover effects, but few studies have explored the effects on partners.

**Methods:**

Dyads included an index participant with BMI ≥ 27 kg/m^2^ and a cohabitating partner with BMI ≥ 18 kg/m^2^ randomized to a participant‐only (*N* = 116) or partner‐assisted (*N* = 115) intervention. All index participants received an evidence‐based group weight management program. Partners in the partner‐assisted group attended half of the sessions but were not required to make behavioral changes/lose weight. Linear mixed models compared changes in partner weight and self‐reported caloric intake between groups at 24 months.

**Results:**

Most partners identified as male (63%) and White (88%). Mean baseline weight and BMI were 98 kg (SD 28.1) and 32 kg/m^2^ (SD 7.8), respectively. At 24 months, partners in the partner‐assisted versus participant‐only group lost more weight (−2.5 kg vs. −0.4 kg; estimated mean difference: −2.0 kg [95% CI −3.5, −0.6]). There were no differences in caloric change (partner‐assisted: −367 kcal, participant‐only: −196 kcal; estimated mean difference: −171 kcal [95% CI −358, 17]).

**Conclusions:**

Involving romantic partners is an efficient weight management approach that generates meaningful long‐term weight loss for cohabitating partners despite them not being the direct targets for weight loss.

**Trial Registration:**

Clinicaltrials.gov NCT03801174

## 1 Introduction

Social relationships influence weight and weight‐related behaviors, including physical activity and healthy eating. Weight clusters within social groups, such that individuals with a higher body mass index (BMI) are more likely to have social network members (e.g., romantic partners, family, friends) who also have a higher BMI [1, 2]. Health behaviors and weight can also “spread” through social networks: when one person in the network increases their physical activity or gains weight, their spouses, family, and friends are more likely to follow a similar trajectory [3, 4]. The influence of social relationships has also been observed within the context of behavioral weight management programs and interventions, which involve changing eating and physical activity habits, combined with behavior change strategies (e.g., goal setting, self‐monitoring) to reduce weight or prevent weight gain [5, 6].

Some studies have demonstrated that one spouse's participation in a behavioral weight management intervention can lead to improvements in behaviors and weight in the spouse who does not participate in the intervention in any capacity (i.e., is *untreated*), suggesting a “ripple effect” that can promote weight loss within a social network absent of any involvement in treatment [7, 8, 9, 10]. However, it is less well understood whether formally including partners in treatment can lead to greater benefits, both for the partner and the person who is the focus of the intervention (index participant). This secondary analysis of the Partner2Lose trial sought to explore the effect of partner participation in a behavioral weight management intervention on their own outcomes [11]. The trial was designed to compare the long‐term (24‐month) efficacy of a partner‐assisted intervention, wherein cohabitating romantic partners were formally included in an evidence‐based weight management program with index participants (intervention group) versus a participant‐only intervention, wherein partners did not participate (control group).

The partner‐assisted intervention was based on the interdependence model of communal coping, which occurs when a one or more individuals view a stressor as a joint problem (shared appraisal) versus an individual problem, and “activate a process of shared or collaborative coping” and cooperate to adopt behavior change [11, 12, 13]. To engender communal coping, the intervention drew on the principles of cognitive behavioral couples therapy (CBCT) to enhance communication skills, particularly sharing thoughts and feelings and joint problem solving/decision‐making [14]. At 24 months, index participants in both groups lost a similar and modest amount of weight, and there were no differences in daily calories or steps [15]. In this secondary analysis of partner data from the Partner2Lose trial, differences were examined between intervention and control group partners in 24‐month changes in weight and self‐reported dietary intake. The hypothesis was that partners in the intervention (partner‐assisted) group, compared to the control (participant‐only) group, would have greater improvements in these outcomes.

## 2 Materials & Methods

### 2.1 Design

This was a two‐group, parallel, randomized controlled trial among dyads, including an index participant meeting criteria for behavioral weight management and a cohabitating partner [6]. Across five cohorts, dyads were randomly assigned with equal probability to the participant‐only or partner‐assisted arm. Prior to March 2020, weight management intervention sessions and outcome assessment were conducted in person, which transitioned to virtual after the onset of the COVID‐19 pandemic. Index participant outcomes have been reported [15]; partner outcomes are the focus of this study. The Health Sciences Institutional Review Board of the University of Wisconsin School of Medicine & Public Health approved the study (#2018–1400).

### 2.2 Participants

Detailed eligibility criteria have been reported [11]. Briefly, index participant inclusion criteria were: aged 18–74 years; BMI of 27–29.9 kg/m^2^ with at least one obesity‐related comorbidity or BMI ≥ 30 kg/m^2^; cohabitation and daily contact with a spouse or romantic partner; English speaking; and possession of a smart phone and e‐mail address. Exclusion criteria included medical comorbidities influencing weight or making participation unsafe. Partners had to be aged 18 or older and possess a smart phone and email address. Partners were excluded if they had BMI< 18.5 kg/m^2^, which served as a proxy for partners who may be unable to support index participants' decreased calorie intake; severely impaired hearing; or health conditions that would limit the ability to provide support. Both dyad members had to be available for at least one (of two) group meeting times for each arm to be eligible.

Detailed recruitment procedures have been reported [11]. Potential participants completed a preliminary eligibility screener online, and for those eligible, study staff conducted an additional phone screening and scheduled eligible dyads for a baseline visit (in‐person for cohorts 1–3 and virtually for cohorts 4 and 5). The baseline visit included a final eligibility determination, including weight measurement on a study scale, informed consent process, completion of baseline measures, and randomization.

### 2.3 Interventions

Index participants in both arms received a manualized evidence‐based behavioral weight‐management program delivered by one of two trained registered dieticians (RDs) [11]. The program involved a 6‐month weight loss component, a 12‐month weight loss maintenance component (months 6–18), and a 6‐month no intervention component (months 19–24). The weight loss phase included 13, 90‐min, every‐other‐week sessions on nutrition education, behavior change strategies, and physical activity instruction [16]. Details of session content have been reported previously [11, 16]. Index participants identified a SMART (specific, measurable, attainable, relevant, and timebound) goal during each session, focusing on one of several discrete options related to the session's content (e.g., for the session on grocery shopping, options included produce, shopping list, whole grains, and healthy snacks) [16]. Goal selections were recorded by study staff at the end of each session and used to send tailored text messages to index participants in both arms throughout the 24‐month study.

Partners in the partner‐assisted arm attended six of 13 weight loss group sessions with the index participant (sessions 2, 3, 5, 7, 9, and 11). Partners attended a subset, rather than all, group sessions to reduce burden and to allow opportunities for index participants to discuss potential challenges with partner involvement. Partners were informed that they could work on their own behavior change and weight loss, if desired, but were not required to because elevated BMI was not a component of partner eligibility criteria. In group sessions attended by the partner (hereafter, joint sessions), all attendees received the same weight management and maintenance content. In the first joint session, the RD reviewed the partner's role in the study and highlighted the intervention's focus on the two key communication skills of joint problem‐solving/decision‐making and sharing thoughts and feelings [14]. The first two joint sessions covered steps for shared decision‐making and guidelines for speaking and listening when sharing thoughts and feelings. All joint sessions included breakout activities, wherein each dyad completed guided exercises to practice communication skills, discuss challenges, and identify behaviors the partner could enact to support the index participant.

In joint sessions, dyads identified a partner support plan to assist the index participant in achieving their specified goal. Support plans were selected from a list of seven options that were consistent across the study, including (1) do it together, (2) provide gentle reminders, (3) praise your partner, (4) remember the long game, (5) check‐in with your partner, (6) be mindful of how your choices affect your partner's goals, and (7) talk with your partner to develop a support plan at home. In sessions only attended by index participants, the index participant identified their goal and desired partner support plan, and the RD encouraged them to share this information with their partner. Partner support plans were recorded by the study team at the end of each session, which informed tailored text messages to partners throughout the study, including reminders to partners about the key session messages, the index participant's goal, and the partner support plan selected at the last session. Details on the technical development were reported previously [16].

The weight maintenance phase in both arms began in month seven and involved three monthly group sessions and nine one‐on‐one calls with the RD, delivered monthly and then bimonthly. Group sessions addressed reasons for weight regain, skills and habits for weight loss maintenance, and the role of physical activity in maintenance. Phone calls addressed the index participant's satisfaction with weight loss outcomes, weight self‐monitoring, relapse prevention, and social support. In the partner‐assisted arm, partners attended all three group sessions and five of the nine maintenance calls. In joint maintenance sessions, breakout activities covered applying communication skills to weight maintenance. Joint maintenance phone calls elicited the partner's reflections on the participant's satisfaction with outcomes, including those experienced by the couple. Dyads also developed support plans for relapse prevention and self‐monitoring during calls.

Partners in the participant‐only group did not participate in any of the weight management intervention sessions or calls. Dyads in this group were offered the opportunity to attend two communication skills sessions following completion of 24‐month outcome collection.

### 2.4 Randomization and Masking

A statistician used varying block sizes of four or six to generate the randomization scheme, which was uploaded to a secure online study database in Research Electronic Data Capture (REDCap) [17]. Randomization was stratified by index participant sex (male/female) and BMI (< 35 kg/m^2^ vs. ≥ 35 kg/m^2^) and partner BMI (< 27 kg/m^2^ vs. ≥ 27 kg/m^2^, reflecting the cutoff when behavioral weight management is recommended). Dyads were randomized at the end of the baseline visit, with a session time rather than group assignment displayed to preserve staff masking for outcome assessments. Group assignment was revealed to the participants at the first session, which only index participants attended.

### 2.5 Outcomes

During the baseline visit, study staff collected self‐reported demographic information from index participants and partners, including age, race, ethnicity, sex assigned at birth, gender identity, marital status, education, work status, financial stress, health insurance coverage, tobacco use, number of previous weight loss attempts, and relationship closeness with the Unidimensional Relationship Closeness Scale [18].

Partners completed follow‐up assessments at months 6, 12, 18, and 24, receiving $40 for assessments at months 6, 12, and 18. Cohorts 1‐4 received $60 at month 24, and cohort 5 received $70 to enhance retention. Prior to the pandemic, study staff obtained in‐person height from a stadiometer and weight from a calibrated Tanita scale at the baseline visit, with in‐person weights at follow‐up timepoints. Values were double‐entered into REDCap with discrepancies resolved before saving data. After pandemic onset, all dyads were mailed a bathroom scale and could either (1) be weighed in person (if permitted by stay‐at‐home orders) or (2) submit a photo that included their feet and weight on the scale and enter their weight into a REDCap survey sent over text message and email by study staff. A study team member reviewed photos and verified self‐reported weights.

To assess dietary intake, partners also completed the Automated Self‐Administered Dietary Assessment 24‐h Dietary Assessment Tool (ASA24) at each timepoint with a 6‐month recall period [19, 20]. Partners received an email or text message prompting them to enter one weekday and one weekend day during the assessment window, with reminders and a study‐created instructional video demonstrating how to complete the ASA24 for those who did not enter data within 24 hours.

### 2.6 Sample Size

The target sample size of 230 dyads (115 per treatment group) for the trial was based on detecting a clinically significant between‐group difference in weight change from baseline to 24 months of 2.5 kg for index participants [11].

### 2.7 Statistical Analysis

Baseline demographic characteristics of partners were examined overall and by treatment group, including means and standard deviations (SD) or interquartile range (IQR) for continuous variables and numbers and percentages for categorical variables. The distribution of partner support plans selected at each session and across all sessions was examined descriptively for the partner‐assisted arm. Outcomes measured at months 0, 6, 12, 18, and 24 were modeled with a linear mixed model (LMM) against the discrete timepoints and interactions with the treatment group and partner as a random effect, where the baseline group means were constrained to be the same owing to randomization [21]. Fixed covariates in the LMM included randomization strata and recruitment cohort. Analyses followed intention‐to‐treat principles. The percentage of partners achieving clinically significant weight loss from baseline of at least 5% in each group was calculated without inferential tests. Multiple Imputation by Chained Equations (MICE) was employed to impute missing data (e.g., weight outcomes), using partner data from all timepoints (including all observed weights) and 20 imputations [22].

Baseline characteristics and follow‐up weights among partners with and without 24‐month outcomes were examined separately by treatment group to explore the potential for non‐response bias. Because prior partner‐assisted weight loss studies have found differences in treatment effects by sex, in exploratory analyses models were fit for the weight outcome separately by partner sex (male/female) [23]. A sensitivity analysis for the weight outcome restricted to partners with BMI ≥ 27 kg/m^2^, the threshold at which behavioral weight management is recommended [6]. Finally, because partner baseline weights differed between groups by chance, despite randomization, models were estimated without constraining baseline weight group means to be equal, both with and without applying multiple imputation. All analyses used a significance level of *p* ≤ 0.05 and were performed in R version 4.0.2 (R Foundation for Statistical Computing, Vienna, Austria).

## 3 Results

**FIGURE 1:**
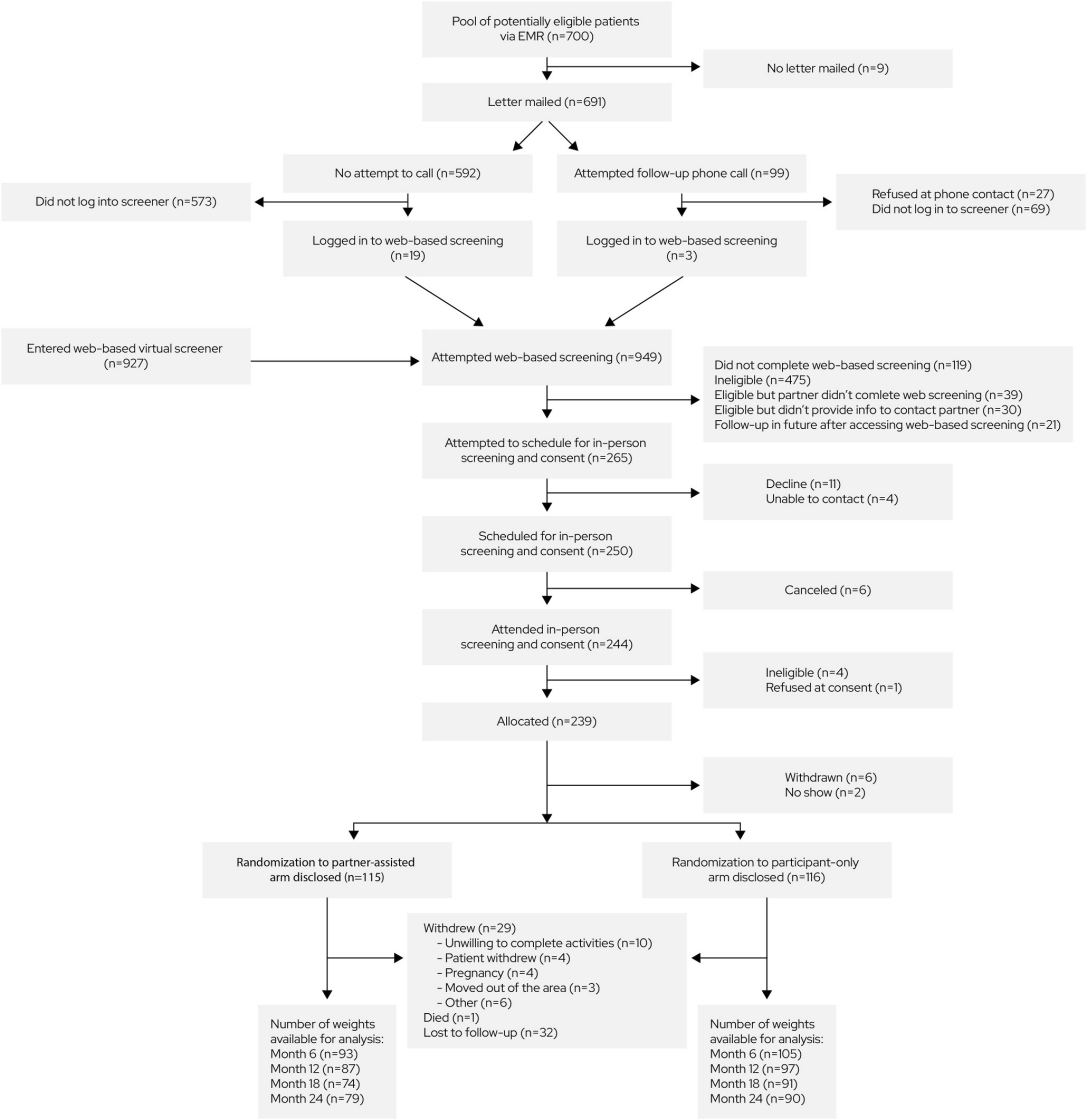
CONSORT study diagram for partners.

Recruitment outcomes have been reported and are displayed in Figure 1 [15]. One hundred fifteen dyads were allocated to the partner‐assisted group and 116 to the participant‐only group. Across treatment groups, the characteristics of partners were generally similar (Table 1). Partner average age was 48.3 years; 88.0% were White and 95.2% were non‐Hispanic; 37.7% were assigned female sex at birth; and 99.2% were cisgender. Most partnerships reflected married couples (87.4%), with 61.9% involving a female index participant/male partner. Partners reported relatively high socioeconomic status (SES), based on education, employment, health insurance, and financial situation. Average baseline weight among partners was 97.6 kg (SD 28.1), and average BMI was 32.0 kg/m^2^ (SD 7.8), with more than a quarter having a BMI above 35 kg/m^2^. Partners in the partner‐assisted group had a higher baseline weight than those in the participant‐only group (100.2 vs. 94.9 kg), and a larger percentage had BMI ≥ 35 kg/m^2^ (33.9% vs. 21.5%). Across groups, average self‐reported baseline daily caloric intake was 2212.1 kcal (SD 798.4).

**TABLE 1:** Characteristics of partners, overall and by treatment group^a^.

Characteristic	Overall (*n* = 231)	Partner‐assisted (*n* = 115)	Participant‐only (*n* = 116)
Partnership identities (Participant: Partner)^b^			
Female: Male	143 (61.9%)	72 (62.6%)	71 (61.2%)
Male: Female	72 (31.2%)	35 (30.4%)	37 (31.9%)
Female: Female	11 (4.8%)	4 (3.5%)	7 (6.0%)
Male: Male	2 (0.9%)	2 (1.7%)	0 (0.0%)
Female: Multi‐gender	1 (0.4%)	0 (0.00%)	1 (0.9%)
Genderqueer: Genderqueer	1 (0.4%)	1 (0.9%)	0 (0.0%)
Multi‐gender: Female	1 (0.4%)	1 (0.9%)	0 (0.0%)
Married partnership	202 (87.4%)	97 (84.4%)	105 (90.5%)
Assigned female sex at birth^c^	87 (37.7%)	42 (36.5%)	45 (38.8%)
Gender identity			
Female	84 (36.4%)	40 (34.8%)	44 (37.9%)
Male	145 (62.8%)	74 (64.4%)	71 (61.2%)
Genderqueer	1 (0.4%)	1 (0.9%)	0 (0.0%)
Multi‐gender	1 (0.4%)	0 (0.00%)	1 (0.9%)
Age, M (SD)	48.3 (12.1)	48.4 (12.5)	48.1 (11.8)
Non‐Hispanic/Latino	218 (95.2%)	109 (95.6%)	109 (94.8%)
Race			
White	198 (88.0%)	99 (89.2%)	99 (86.8%)
Black or African American	12 (5.3%)	9 (8.1%)	3 (2.6%)
Asian	10 (4.4%)	2 (1.8%)	8 (7.0%)
American Indian or Alaska Native	1 (0.4%)	0 (0.0%)	1 (0.9%)
Multiracial	4 (1.8%)	1 (0.9%)	3 (2.6%)
Education			
High school graduate or less	10 (4.3%)	8 (7.0%)	2 (1.7%)
Trade/technical/or vocational school	5 (2.2%)	1 (0.9%)	4 (3.4%)
Some college, no degree	18 (7.8%)	7 (6.1%)	11 (9.5%)
Associate degree	23 (10.0%)	15 (13.0%)	8 (6.9%)
Bachelor's degree	84 (36.4%)	36 (31.3%)	48 (41.4%)
Post‐graduate work or degree	80 (34.6%)	38 (33.0%)	42 (36.2%)
Employed full‐time	162 (70.4%)	85 (74.6%)	77 (66.4%)
Financial status			
Poor/just getting along	26 (11.3%)	17 (14.8%)	9 (7.8%)
Prosperous	13 (5.6%)	5 (4.4%)	8 (6.9%)
Reasonably comfortable	119 (51.5%)	56 (48.7%)	63 (54.3%)
Very comfortable	73 (31.6%)	37 (32.2%)	36 (31.0%)
Financial situation			
Difficulty paying bills	0 (0.0%)	0 (0.0%)	0 (0.0%)
Enough to pay bills after cut back	12 (5.2%)	10 (8.7%)	2 (1.7%)
Little to spare for special things	68 (29.6%)	35 (30.4%)	33 (28.7%)
Enough for special things	150 (65.2%)	70 (60.9%)	80 (69.6%)
Health insurance			
Employer	200 (88.1%)	101 (89.4%)	99 (86.8%)
Self‐purchased	14 (6.2%)	6 (5.3%)	8 (7.0%)
Medicare	26 (11.5%)	12 (10.6%)	14 (12.3%)
Medicaid	3 (1.3%)	2 (1.8%)	1 (0.9%)
Military	2 (0.9%)	1 (0.9%)	1 (0.9%)
VA	4 (1.8%)	1 (0.9%)	3 (2.6%)
Currently use nicotine	13 (5.6%)	10 (8.7%)	3 (2.6%)
Attempted weight loss previously	175 (76.1%)	86 (74.8%)	89 (77.4%)
Number of attempts, median (IQR)	3.0 (2.0–5.0)	3.0 (2.0–5.0)	3.0 (2.0–5.0)
BMI kg/m^2^, M (SD)	32.0 (7.8)	32.7 (8.4)	31.2 (7.0)
BMI < 35 kg/m^2^	167 (72.3%)	76 (66.1%)	91 (78.5%)
BMI < 27 kg/m^2^	66 (28.6%)	32 (27.8%)	34 (29.3%)
Weight, kg, M (SD)	97.6 (28.1)	100.2 (30.1)	94.9 (25.9)
Daily caloric intake, kcal, M (SD)^d^	2212.1 (798.4)	2234.6 (875.5)	2189.6 (716.3)
Relationship closeness^e^	6.3 (0.7)	6.3 (0.7)	6.3 (0.7)

^a^ All numbers are *n* (%) unless indicated otherwise; percents may not add to 100% due to missing data.

^b^ Partnership identities are based on gender identity.

^c^ Sex assigned at birth was used for stratification of index participants and partners.

^d^ Daily caloric intake was estimated based on responses to the Automated Self‐Administered 24‐Hour Dietary Assessment Tool.

^e^ Relationship closeness was measured using the 11‐item Unidimensional Relationship Closeness Scale.

In the partner‐assisted group, partners attended a median of 6 (of 9) planned sessions (range 0–9, IQR = 4, 8) and a median of 4 (of 5) maintenance phone calls (range 0–5, IQR = 3, 5). For nearly all sessions, the most selected partner support plans were “do it together” and “talk with your partner to develop a support plan at home” (Table 2). At 24 months, a greater proportion of partners in the participant‐only group provided outcomes compared with the partner‐assisted group: 77.6% and 68.7% for weight and 63.8% and 57.4% for caloric intake. Baseline characteristics among those with and without 24‐month weights were similar (data not shown). Among those missing 24‐month weights, average weights at all earlier timepoints were higher than among those with 24‐month outcomes (Supporting Information S1: Figure S1), and they lost less weight at their last observable outcome measurement than those with 24‐month outcomes (Supporting Information S1: Figure S2).

**TABLE 2:** Distribution of partner support plans selected in each weight loss initiation session and across all sessions in the partner‐assisted group.

Session number and content	Be mindful of how your choices affect your partner		Check in with your partner		Do it together		Praise your partner		Provide gentle reminders		Remember the long game		Talk with your partner to develop a support plan at home	
	*N*	%	*N*	%	*N*	%	*N*	%	*N*	%	*N*	%	*N*	%
2: Interpreting food label/SMART goals^a^	10	9.2	13	11.9	46	42.2	11	10.1	10	9.2	0	0.0	19	17.4
3: Importance of tracking^a^	3	2.8	18	16.8	41	38.3	10	9.3	11	10.3	5	4.7	19	17.8
4: Grocery shopping	10	9.0	15	13.5	39	35.1	14	12.6	9	8.1	4	3.6	20	18.0
5: Meal planning^a^	6	5.5	18	16.5	46	42.2	7	6.4	5	4.6	9	8.3	18	16.5
6: Healthy cooking	3	2.7	13	11.7	38	34.2	9	8.1	2	1.8	6	5.4	40	36.0
7: Dining out^a^	9	8.7	14	13.6	37	35.9	8	7.8	11	10.7	4	3.9	20	19.4
8: Dining out: advanced	11	12.0	8	8.7	33	35.9	8	8.7	15	16.3	5	5.4	12	13.0
9: Physical activity^a^	1	1.0	19	19.0	34	34.0	11	11.0	9	9.0	2	2.0	24	24.0
10: Eating more fruits and vegetables	7	7.1	12	12.1	35	35.4	6	6.1	7	7.1	6	6.1	26	26.3
11: Mindful eating^a^	11	11.0	10	10.0	31	31.0	10	10.0	11	11.0	2	2.0	25	25.0
12: Emotional eating	13	13.4	11	11.3	16	16.5	12	12.4	9	9.3	12	12.4	24	24.7
All sessions combined	84	7.4	151	13.3	396	34.8	106	9.3	99	8.7	55	4.8	247	21.7

^a^ Session attended by partner.

**FIGURE 2:**
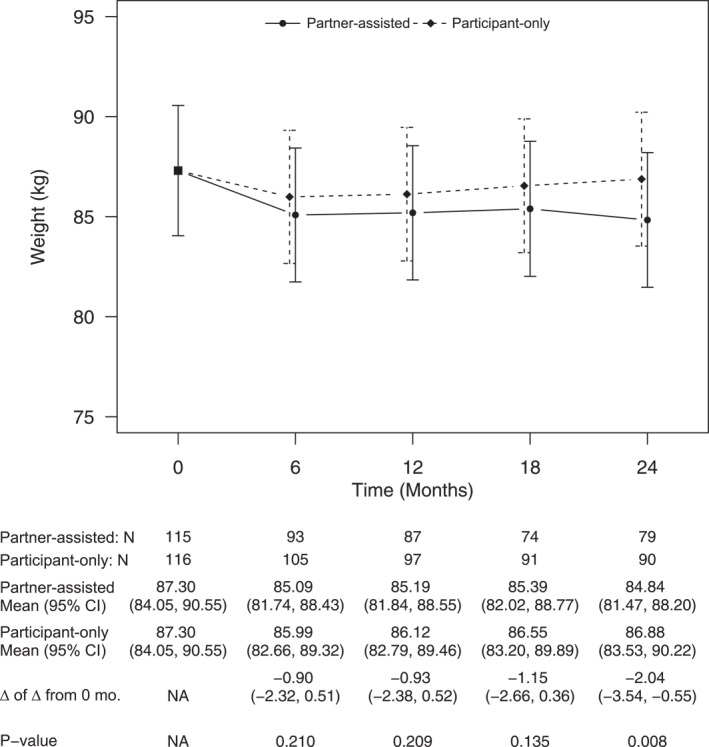
Model estimated partner weights, differences in weights, and associated 95% confidence intervals, by treatment group and timepoint.

**FIGURE 3:**
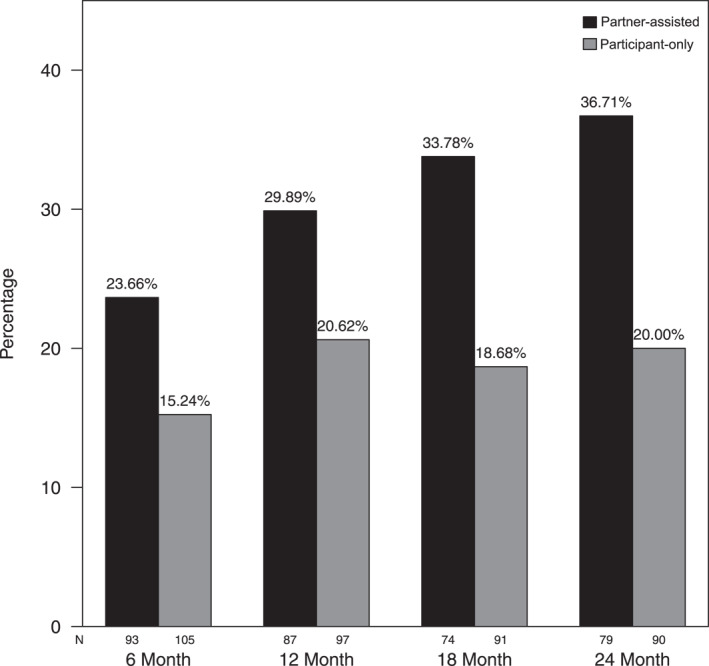
Proportion of partners achieving 5% weight loss from baseline, by treatment group and timepoint.

Average weight change at 24 months was −0.4 kg in the participant‐only group and −2.5 kg in the partner‐assisted group, with an adjusted mean difference of −2.0 kg (95% confidence interval [CI] −3.5, −0.6; *p* = 0.008; Figure 2), indicating greater weight loss in the partner‐assisted group. At all prior timepoints, differences between groups were less pronounced, and none reached statistical significance (6 months: −0.9, 95% CI −2.3, 0.5; 12 months: −0.9, 95% CI −2.4, 0.5; 18 months: −1.2, 95% CI −2.7, 0.4). In analyses employing multiple imputation, differences between groups were generally larger (estimated mean difference at 24 months = −2.7 kg; 95% CI −5.1, −0.3; *p*‐value = 0.03). At all follow‐up timepoints, a larger percentage of partners in the partner‐assisted group, compared to the participant‐only group, achieved at least 5% weight loss from baseline: 24% versus 15% at 6 months, 30% versus 21% at 12 months, 34% versus 19% at 18 months, and 37% versus 20% at 24 months (Figure 3).

Differences between treatment groups were also larger in sensitivity analyses restricting to the 71% of partners with baseline BMI ≥ 27 kg/m^2^ (estimated mean difference = −2.9; 95% CI −4.8, −1.0; *p* = 0.003). In analyses stratified by sex assigned at birth, differences were similar in magnitude (males: estimated mean difference = −2.1; 95% CI −4.2, −0.1; *p*‐value = 0.04; females: estimated mean difference = −1.9; 95% CI −4.0, −0.3; *p*‐value = 0.09). In sensitivity analyses where baseline group mean weights were not constrained to be equal, findings were consistent and slightly more pronounced than when constrained (complete case analysis: estimated mean difference at 24 months = −2.2 kg, 95% CI −3.7, −0.7, *p* = 0.004; multiple imputation: estimated mean difference at 24 months = −2.9 kg, 95% CI −5.0, −0.9, *p* = 0.005).

There were no differences between groups in baseline to 24‐month change in caloric intake (estimated mean difference = −171 kcal; 95% CI −358, 17; *p*‐value = 0.07; Supporting Information S1: Figure S3). Results were similar when restricted to those with BMI ≥ 27 kg/m^2^ (estimated mean difference = −143 kcal, 95% CI −375, 89, *p* = 0.23).

## 4 Discussion

This secondary analysis from the Partner2Lose randomized controlled trial explored the effects of a partner‐assisted vs. participant‐only intervention on partner weight and caloric intake. Partners in the partner‐assisted group lost 2.0 kg more than partners in the participant‐only group and were more likely to achieve 5% weight loss, even though their weight‐related behaviors and weight were not targets of the intervention. Effects were more pronounced among partners with baseline BMI ≥ 27 kg/m^2^ for whom behavioral weight management is recommended by current guidelines [6]. Importantly, these effects were observed despite partners receiving a lower dose of the intervention and attending only a subset of sessions and calls with index participants. There were no between‐group differences in self‐reported caloric intake.

There are two ways of involving partners in weight‐management treatment: (1) in a support role without requiring that the partner have the targeted disease/health condition or make behavior changes (partner‐assisted intervention) and (2) as a co‐target of intervention with the targeted disease/health condition and intent to make behavior changes (disease‐oriented intervention) [24]. In contrast to the study reported herein, most prior reports of partner effects in dyadic weight management interventions reflect disease‐oriented approaches, where partners also have elevated BMIs, receive weight management education, and are encouraged to change behaviors and lose weight along with index participants. Among the few disease‐oriented weight management interventions that report partner outcomes, partners in the disease‐oriented group lost more weight than partners in the participant‐only group who were not involved in treatment, and within‐group weight loss was slightly larger than that observed in Partner2Lose [23, 25]. The larger weight loss achieved among involved partners in those studies versus Partner2Lose may be explained by the disease‐oriented compared to partner‐assisted approaches; greater intervention frequency for index participants and partners in the disease‐oriented interventions; and, for one disease‐oriented intervention, home environment modifications that could have impacted spousal partners (e.g., regular pantry clean outs; provision of cookbooks, healthy recipe magazines, portion‐controlled dishware; provision of treadmill or stationary bicycle, resistance bands, and exercise videotape) [23].

In Partner2Lose, although partners in the partner‐assisted group were not explicitly encouraged to make weight‐related behavioral changes or lose weight, they received information on physical activity, diet, weight‐related behavioral modifications, as well as communication skills training to enhance their ability to support the index participant in their behavior change goals. Partners achieved an average *within‐group* weight loss of 2.5 kg even though they only attended a median of six sessions and four phone calls, demonstrating benefits at a relatively small dose. Benefits observed among partners in the partner‐assisted group may also be attributable to providing support to index participants. A prior study found that both receiving and providing autonomy support that is non‐judgmental, empathic, and responsive in the context of a dyadic weight‐management intervention was associated with greater sustained weight loss [26]. Although autonomy support and provided (vs. received) support were not measured in Partner2Lose, the partner support plans and communication skills developed in the partner‐assisted group may have enhanced partners' provision of autonomy support to index participants, contributing to partner weight loss. Findings contrast with the few studies testing partner‐assisted interventions to increase physical activity. Several of those studies compared action planning (forming intentions and plans for when, where, and how a behavior will be carried out) or action control (ongoing monitoring and self‐correction process to maintain plan/goals) interventions, in which the partner's role was to support the index participant to increase physical activity. Compared to participant‐only or control interventions, all but one study [27] reported no or unsustained physical activity benefits among partners in the partner‐assisted group [28, 29, 30]. Notably, these interventions were typically very brief (e.g., one involved only a single session), which may limit the effects observed in both index participants and partners, in contrast to Partner2Lose, which required significantly greater participation for the dyad.

The findings of minimal weight loss among partners of index participants assigned to the participant‐only group contrast somewhat with several prior studies examining the ripple effect of behavioral weight management programs on *untreated* partners. Studies using data from clinical trials and commercial weight management programs have reported weight loss ranging from 1 to 2.4 kg among partners, typically spouses, who did not actively participate in the weight management programming or intervention in any way [8, 9, 10, 23]. However, untreated partners in the Partner2Lose participant‐only group lost < 0.5 kg on average. This discrepancy may be partially explained by the greater weight loss achieved by index participants in prior studies compared with Partner2Lose, suggesting greater changes to the home environment, eating habits, and/or behavioral modeling that positively contributed to partner weight loss. This is consistent with a re‐analysis of Gorin et al. exploring the dyadic dynamics of the ripple effect, which revealed that untreated spouses lost more weight when index participants had high (vs. low) reductions in energy and fat intake [23, 31].

Findings herein should be interpreted considering the strengths and limitations of the study. Prior studies have predominantly examined ripple effects on untreated partners not actively engaged in the intervention, either as co‐targets or supports for index participants. This study enabled exploration of ripple effects in untreated partners (partners in the participant‐only group) as well as partners who were included in the intervention to support weight loss goals of index participants (partners in the partner‐assisted group) and to compare partner effects between these groups. The study also benefits from the two‐year follow‐up, allowing for examination of weight loss maintenance, whereas most dyadic weight‐management trials assess outcomes at 12 months or less. Limitations of the study include a relatively homogenous study population of predominantly non‐Hispanic White, high SES, heterosexual, cisgender dyads composed of female index participants and male partners. This overrepresentation of certain groups is common in behavioral weight‐management trials. Better representation of other populations is needed to explore how weight management interventions generally, and partner‐assisted interventions specifically, perform among these groups, including racialized populations, men, and individuals with low incomes.

An additional limitation is the significant drop out in the study, with only 73% of partners providing weight outcomes at 24 months, similar to the 74% of index participants, which may be due to the concurrent COVID‐19 pandemic [15]. Partners without 24‐month outcomes had higher weights and lost less weight at prior timepoints than those with 24‐month data, which could lead to bias in the complete case analysis. Multiple imputation was used to address missing data, which accounted for all prior observed weights and other variables associated with missingness and 24‐month outcomes, and led to more pronounced differences in partner weight loss outcomes between treatment groups. The possibility exists that the data were missing not at random, such that multiple imputation results may still be biased. The interpretation of findings is bolstered by the consistency of results across analyses, including those with and without imputation and those with and without constraining baseline means to be equal. Another limitation is that physical activity data among partners was not available because the budget did not accommodate fitness trackers for them, precluding examination of differences between treatment groups in this outcome, which may have contributed to weight loss. Finally, a switch from in‐person to virtual intervention delivery could have impacted results; however, non‐randomized analyses of Partner2Lose data suggest greater attendance for virtual versus in‐person sessions and no differences in effectiveness by modality among index participants [32].

These findings support that participation in behavioral weight management as a supportive partner and not as a target for weight loss can result in clinically meaningful weight loss among partners over time, especially among those with elevated BMI. Partners benefited from the intervention even though they received a lower dose, attending only a subset of sessions and calls. Despite similar weight loss among index participants in both treatment groups, weight loss among partners in the partner‐assisted group suggests that dyadic interventions may be a cost‐effective approach to weight management. Future cost‐effectiveness studies are needed that formally incorporate both the costs of including partners and the weight loss‐related benefits for partners. Considering that weight loss was greater when restricting to partners who also met BMI criteria for behavioral weight management, it may be that disease‐oriented approaches (where the partner is a co‐target of the intervention) are most effective when both the index participant and partner have elevated BMIs. However, an advantage of the partner‐assisted approach is that the index participant can participate regardless of partner BMI. Future research could explore whether, among dyads where both have elevated BMIs, a partner‐assisted or disease‐oriented approach is superior.

## Author Contributions

K.E.G., C.I.V., R.J.S., M.A.L., W.S.Y., S.K.P., and L.S.P. participated in conceptualization. R.J.S., K.L.G., S.J.H., H.M.J., and K.G. participated in data curation. S.J.H. conducted formal analysis. C.I.V., R.J.S., M.A.L., H.M.J., L.M., W.S.Y., and L.S.P. acquired funding. K.G. participated in the investigation. C.I.V., R.J.S., S.J.H., M.A.L., F.E., L.M., K.E.G., W.S.Y. and L.S.P. contributed to methodology. C.I.V. and K.L.G. oversaw project administration. C.I.V. provided resources. R.J.S. developed software. C.I.V. and L.S.P. provided supervision. K.L.G., S.J.H., and K.G. provided data validation. S.J.H. provided visualization. K.E.G. wrote the original draft. All authors reviewed and edited the draft.

## Funding

This study was funded by the US National Institutes of Health, National Institute for Diabetes and Digestive and Kidney Diseases (R01DK111491). Dr. Voils was supported by VA Research Career Scientist award (RCS 14‐443) and Dr. Gray by Career Development Award (CDA 16‐154) from the Health Systems Research service of the Department of Veterans Affairs (VA). The views represented in this article represent those of the authors and not those of the VA or the United States Government.

## Conflicts of Interest

Dr. Yancy has consulted for FoodMinds. Dr. Pabich has consulted for Eli Lilly.

## Supporting information


Supporting Information S1

## Data Availability

The dataset and analytic code generated during the current study are not publicly available but are available from the corresponding author on reasonable request.
