# Supplementary material for: Partner Weight Loss and Diet Outcomes at 24 Months in a Randomized Controlled Trial of a Dyadic Versus Individual Weight Management Intervention
Source: Obes Sci Pract. 2026 Aug 12;12(4):e70178. doi: 10.1002/osp4.70178 (PMC13463752; doi:10.1002/osp4.70178)
Supplement: Supplementary file 1 [file OSP4-12-e70178-s001.pdf]

Partner weight loss and diet outcomes at 24 months in a randomized controlled trial of a dyadic vs. individual weight management intervention

Kristen E. Gray,[1,2] Laura S. Porter,[3] Ryan J. Shaw,[4] Kara L. Gavin,[5] Scott J. Hetzel,[6] Megan A. Lewis,[7] Samantha Pabich,[8] Heather M. Johnson,[9] Felix Elwert,[10] Lu Mao,[6] Katya Garza,[11] William S. Yancy Jr.,[12] and Corrine I. Voils[8,13]

[1]VA Puget Sound Health Care System, Health Services Research & Development, 1660 S. Columbian Way, S-152, Seattle, WA, 98108, USA

[2]Department of Health Systems and Population Health, University of Washington, 3980 15 Ave NE, Box 351621, Seattle, WA, 98195, USA

[3]Department of Psychiatry & Behavioral Sciences, Duke School of Medicine, DUMC Box 102506, Durham, NC, 27710, USA

[4]Duke University School of Nursing, 307 Trent Dr., DUMC 3322, Durham, NC 27710, USA

[5]School of Medicine and Public Health, Department of Medicine, University of Wisconsin – Madison, 610 Walnut St, Madison, WI, 53726-2336, USA

[6]Department of Biostatistics and Medical Informatics, University of Wisconsin – Madison, School of Medicine and Public Health, 207A WARF 610 Walnut St., Madison, WI, 53726, USA

[7]RTI International, Research Triangle Park, 3040 East Cornwallis Rd., Research Triangle Park, P.O. Box 12194, NC, 27709-2194, USA

[8]William S Middleton Memorial Veterans Hospital, 2500 Overlook Terrace (151), Madison, WI, 53705, USA

[9]Baptist Health South Florida/Charles E. Schmidt College of Medicine, Florida Atlantic University, 690 Meadows Road, Boca Raton, FL, 33486, USA

[10]Department of Sociology, Department of Biostatistics and Medical Informatics, Department of Population Health Sciences, University of Wisconsin-Madison, 800 Observatory Dr, Madison, WI, 53706, USA

[11]Department of Kinesiology, University of Wisconsin-Madison School of Education, 285 Med Sci 1300 University Ave, Madison, WI, 53706-1121 USA

[12]Department of Medicine, Duke University School of Medicine, 501 Douglas Street, Durham, NC, 27705, USA

[13]Department of Internal Medicine, Spencer Fox Eccles School of Medicine, University of Utah, 295 Chipeta Way Suite #3, Salt Lake City, UT, 84108, USA

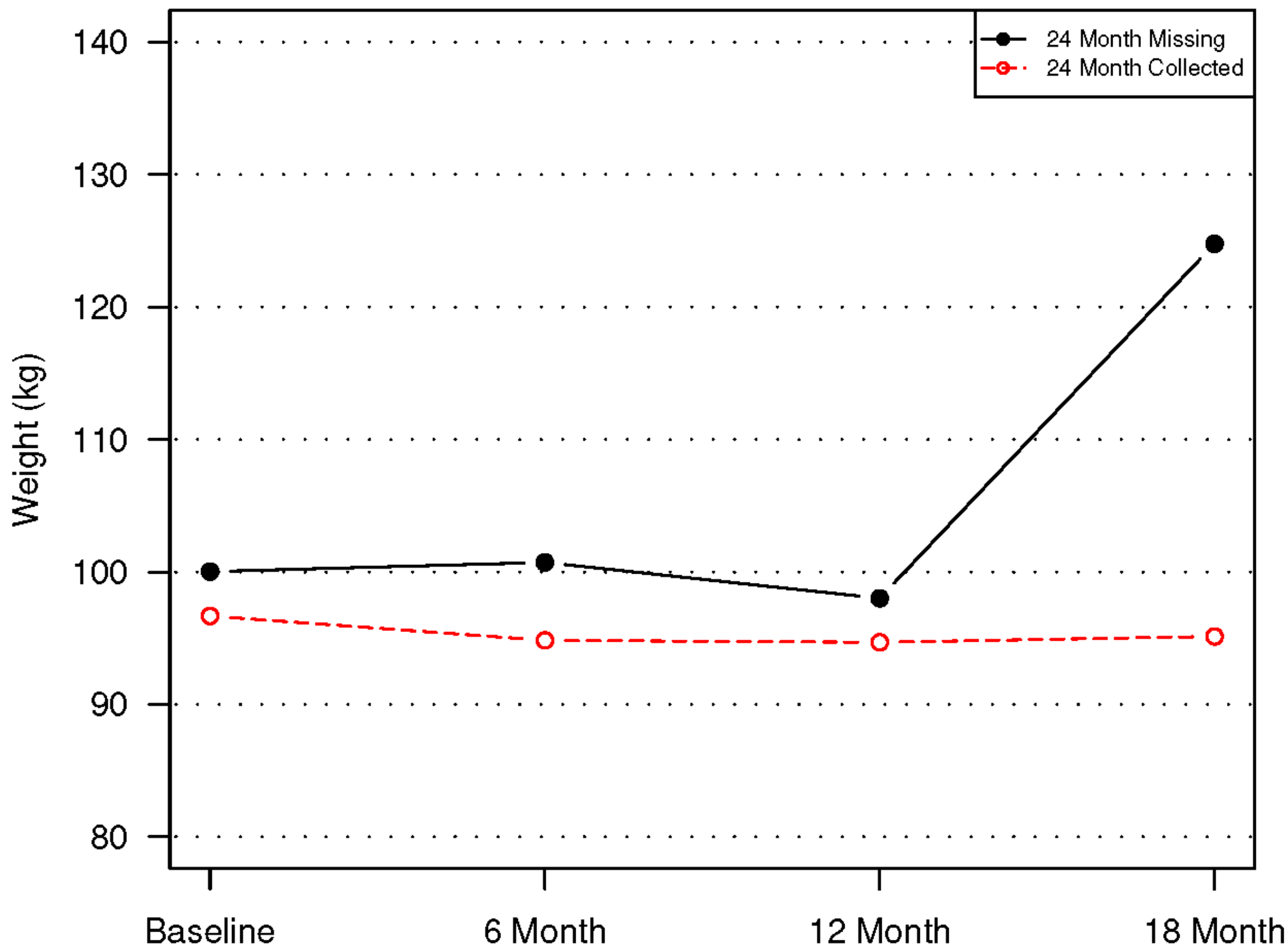

Figure S1. Mean partner weight at each time point, stratified by availability of 24-month weight. At all prior time points, average weights were higher among participants with missing 24-month weights than those with available 24-month weights.

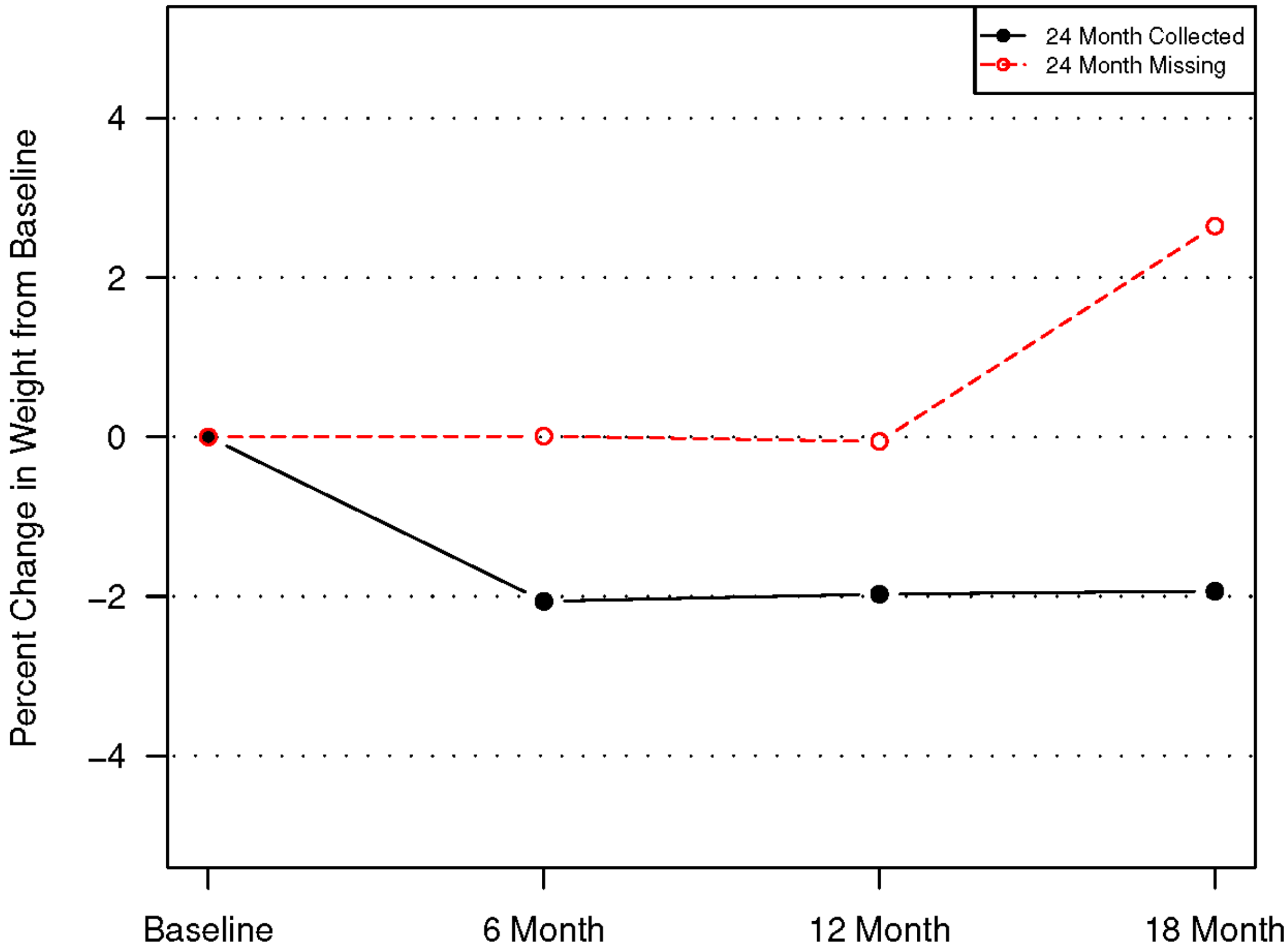

Figure S2. Partner percent weight change from baseline, stratified by availability of 24-month weight.

At prior time points, average partner percent weight loss was greater among those with available 24-month weights than those with missing 24-month weights.

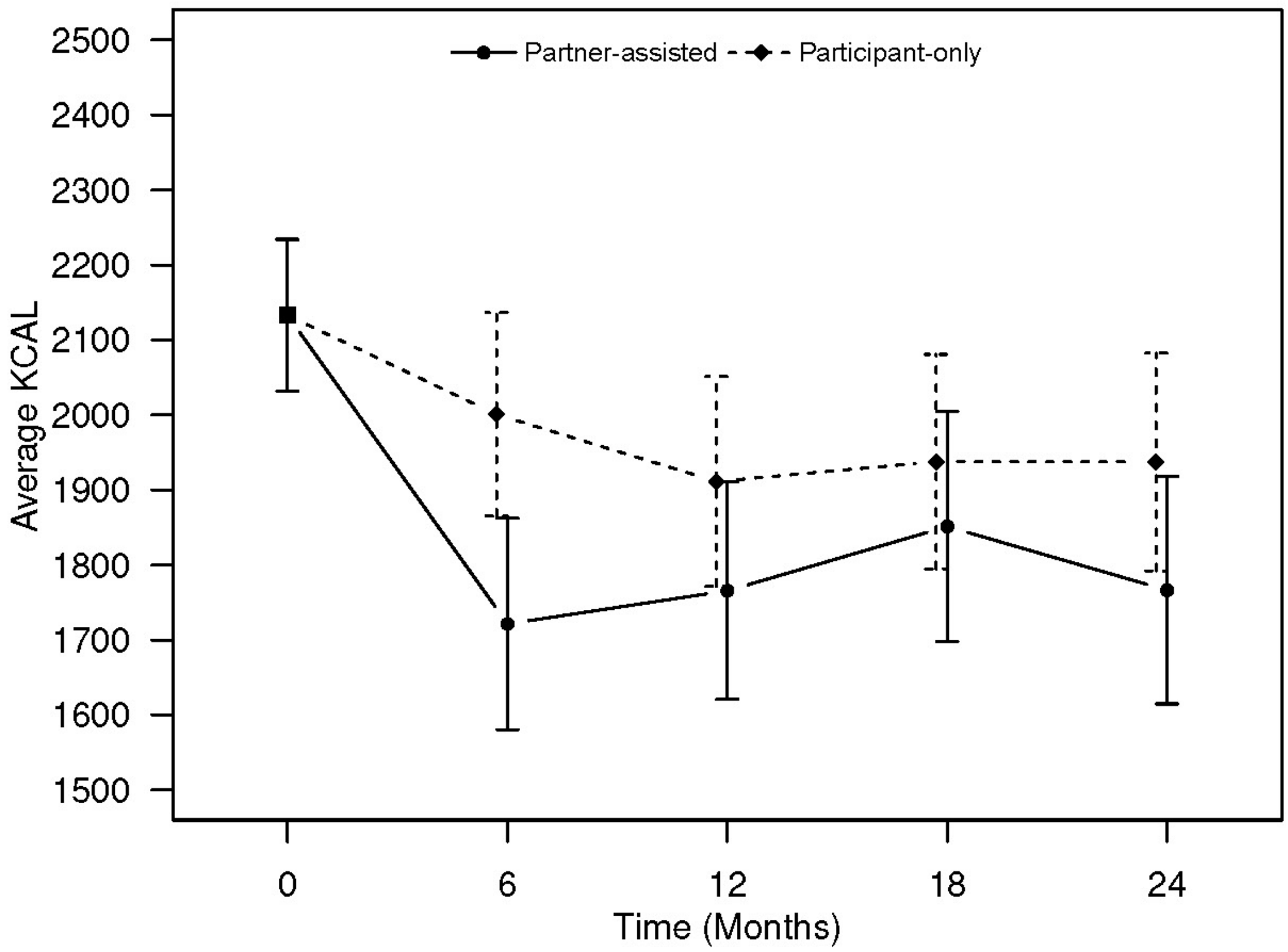

	0	6	12	18	24
Partner-assisted: N	111	83	75	64	66
Participant-only: N	111	90	82	78	74
Partner-assisted Mean (95% CI)	2133 (2032, 2234)	1721 (1581, 1862)	1766 (1621, 1911)	1851 (1698, 2005)	1766 (1615, 1918)
Participant-only Mean (95% CI)	2133 (2032, 2234)	2001 (1865, 2136)	1911 (1771, 2051)	1938 (1795, 2081)	1937 (1792, 2083)
Δ of Δ from 0 mo.	NA	−279 (−450, −108)	−146 (−324, 33)	−87 (−273, 100)	−171 (−358, 17)
P−value	NA	0.001	0.109	0.364	0.074

Figure S3. Model estimated partner calorie intake, differences in calorie intake, and associated 95% confidence intervals, by treatment group and time point. At 24 months, change in caloric intake from baseline was similar among partners in the partner-assisted and participant-only groups.
